# Safety and Efficacy of Adding Dapagliflozin to Furosemide in Type 2 Diabetic Patients With Decompensated Heart Failure and Reduced Ejection Fraction

**DOI:** 10.3389/fcvm.2020.602251

**Published:** 2020-12-07

**Authors:** Ayman Ibrahim, Ramadan Ghaleb, Hossam Mansour, Amr Hanafy, Naggeh M. Mahmoud, Mohamed Abdelfatah Elsharef, Mohamed Kamal Salama, Saud M. Elsaughier, Lobna Abdel-Wahid, Mona Embarek Mohamed, Ahmed K. Ibrahim, Ahmed Abdel-Galeel

**Affiliations:** ^1^Cardiology Department, Aswan University, Aswan, Egypt; ^2^Internal Medicine Department, Assiut University, Assiut, Egypt; ^3^Microbiology and Immunology Department, Faculty of Medicine, Assiut University, Assiut, Egypt; ^4^Community Medicine Department, Assiut University, Assiut, Egypt; ^5^Cardiovascular Medicine Department, Assiut University Heart Hospital, Assiut University, Assiut, Egypt

**Keywords:** dapagliflozin, furosemide, diuretic, heart failure, diabetes mellitus

## Abstract

**Background:** Heart failure is the most common cause of hospitalization in elderly patients. It is likely that many of the mechanisms that contribute to reductions in systolic and diastolic function, seen in diabetic patients, place them at an increased risk of heart failure. Diuretic therapy, especially loop diuretics, is the usual way of managing congestion, particularly in volume-overloaded patients. Little is known about the beneficial effect of dapagliflozin when added to loop diuretics in managing patients with decompensated heart failure.

**Aim:** To assess the effect of the addition of dapagliflozin to furosemide in managing decompensated patient with heart failure and reduced left ventricular ejection fraction in terms of weight loss and dyspnea improvement.

**Patients and Methods:** The study included 100 type 2 diabetic patients who were admitted with decompensated heart failure. The study population was randomly divided into two arms. Serum electrolytes and kidney functions were followed up during their hospital stay.

**Results:** With dapagliflozin, there was a statistically significant difference between the two groups regarding the change in body weight and body mass index. The diuresis parameters including urine output, total fluid loss, and fluid balance also showed a statistically significant difference in favor of the use of dapagliflozin, with no significant change in serum potassium or kidney functions. There was significant improvement in patient-reported dyspnea scores with the use of dapagliflozin.

**Conclusions:** Dapagliflozin may provide a new drug option in the treatment of heart failure especially among vulnerable group of diabetics. It had no remarkable effects on serum potassium level and kidney functions.

**Clinical Trial Registration:** www.ClinicalTrials.gov, identifier: NCT04385589.

## Introduction

Congestion is the most frequent cause of hospitalization in acute decompensated heart failure (ADHF) (1). Diuretic therapy, especially loop diuretics, is the 1st line treatment of congestion, especially in volume-overloaded patients (2). Loop diuretics are heavily protein-bound and secreted into the proximal convoluted tubule. Therefore, adequate dosing with sufficient plasma levels is crucial for their action. They inhibit the Na+/K+/2Cl co-transporter at the ascending loop of Henle, so they have a potent diuretic effect, promoting excretion of sodium and chloride (3).

The DOSE trial studied different dosages and intermittent vs. continuous prescription of loop diuretics in acute heart failure (HF). The study stated that higher dosages were associated with more fluid and weight loss, yet a higher incidence of worsening renal function (WRF) (4).

It is well known that potassium-sparing diuretics reduced both hospitalizations and mortality in patients with chronic HF. However, in patients with ADHF, they are less effective (5). Moreover, diuretic resistance is frequently reported in HF patients (6).

Mechanisms responsible for reductions of systolic and diastolic functions present in diabetic patients might increase the risk of HF (7, 8). Moreover, the association between mortality and HbA1c in diabetic patients with HF is well documented (9). Some studies suggest that diabetes mellitus (DM) is independently associated with a greater risk of death and rehospitalization compared with nondiabetics with HF (10). Epidemiological studies have shown that HF incidence was 2–4 folds higher in people with diabetes compared to those without diabetes (11, 12).

In a retrospective cohort study, sulfonylurea was correlated with increased HF risk when compared with metformin (13). In type 2 diabetic patients with atherosclerotic cardiovascular disease (ASCVD) experiencing myocardial infarction, sitagliptin did not improve the subsequent risk of cardiovascular mortality or HF hospitalization (14). An unexpected finding of the SAVOR-TIMI 53 trial was that the incidence of hospitalization for HF was higher in patients who received saxagliptin compared with the placebo group (15).

The Empagliflozin Cardiovascular Outcome Event Trial in Type 2 Diabetes Mellitus Patients (EMPA-REG OUTCOME), investigated the effects of empagliflozin on cardiovascular (CV) outcomes in patients with T2DM and established atherosclerotic disease, and found a 35% relative risk reduction in HF hospitalization (16). In both the Canagliflozin Cardiovascular Assessment Study (CANVAS) and the Canagliflozin Cardiovascular Assessment Study—Renal (CANVAS-R), canagliflozin significantly reduced HF hospitalization vs. the placebo in diabetic patients with and without a history of HF (17). In the Dapagliflozin Effect on Cardiovascular Events-Thrombolysis in Myocardial Infarction 58 trial (DECLARE-TIMI 58), dapagliflozin significantly reduced the risk of a composite outcome of HF hospitalization or CV death vs. the placebo (18).

Volume contraction, a result of natriuresis and diuresis, has been hypothesized to play a major role in sodium-glucose co-transporter-2 (SGLT2) inhibitor-associated CV benefits (19). In HF patients with reduced ejection fraction (EF), those treated with dapagliflozin had a lower risk of worsening HF or CV mortality than those who received the placebo, regardless of the presence or absence of diabetes (20). The combination of empagliflozin and loop diuretics seems to have synergistic effects on diuresis, without inducing renin-angiotensin-aldosterone system (RAAS) activation. Additionally, it resulted in a significant increase in both urinary sodium concentration, and peak oxygen consumption (21).

The current study aimed at assessing the adjusted effect of adding dapagliflozin to furosemide in managing decompensated HF patients with reduced left ventricular EF in terms of body weight reduction and dyspnea improvement, and also, to assess the subsequent effects on blood sugar level, kidney function and serum electrolytes.

## Patients and Methods

### Patients

**Figure 1:**
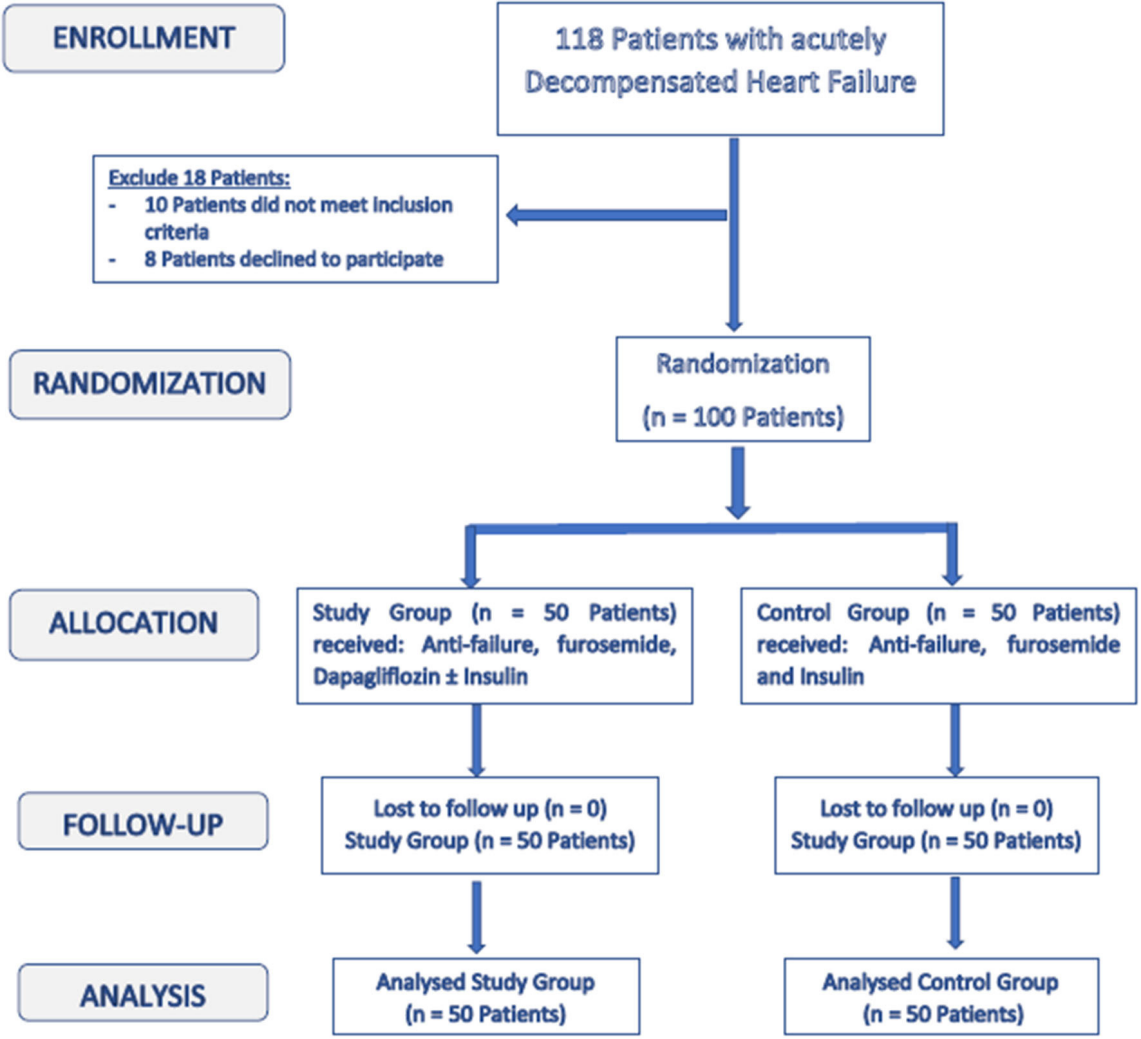
Enrollment and follow up of the study population.

The study included 100 DM patients (type 2) who were admitted to Aswan University Hospital, at the cardiac care unit and Assiut University Heart Hospital, at the critical care unit with decompensated HF. The sample size calculation was carried out using G^*^Power 3 software. A calculated minimum sample of 94 patients with type 2 DM and HF (47 -Group A- and 47 -Group B-) was needed to detect an effect size of 0.3 in the change in weight and body mass index (BMI), with an error probability of 0.05 and 80% power on a two-tailed test. The sample was raised to include 100 patients. Figure 1 is a flowchart of the study population.

Inclusion criteria were age more than 18 years, type 2 diabetic patients with history of chronic HF and had indication for admission to cardiac care unit (decompensated HF). The patients were included as they had at least one symptom (respiratory discomfort or orthopnea) and one clinical sign (peripheral edema, engorged jugular vein, or pulmonary congestion), the patients were already on furosemide for at least 1 month before admission plus other conventional anti-failure treatment, had left ventricular ejection fraction (LVEF) ≤ 40% and there was no prespecified inclusion criterion with respect to HF etiology.

The patients were excluded if they had any of the following: other causes of fluid overload different than HF, marked hyponatremia; sodium level below 125 mmol/l, unstable patients; acute coronary syndrome, cardiogenic shock, patients requiring positive inotropic agents, or renal dialysis, pregnant or breast-feeding, advanced hepatic disease, advanced kidney disease with glomerular filtration rate (GFR) <45 mL/min/1.73 m^2^ and patients with diabetic ketoacidosis.

The study population was randomly divided into two groups:

**Group I (study arm):** included 50 patients who received dapagliflozin alone or with insulin (when needed) for control of blood glucose levels and furosemide plus conventional anti-failure measures.
**Group II (control arm)**: included 50 patients who received insulin for control of blood sugar and furosemide plus other anti-failure measures.

### Methodology

(A) *Group I (Study arm)*: The patients received:
  (a) **Furosemide**: It was administered at doses sufficient to achieve optimal volume status and relieve congestion without inducing an excessively rapid reduction in intravascular volume. Furosemide was given intravenously either by continuous infusion or boluses.
  (b) **Anti-failure treatment**: Angiotensin converting enzyme inhibitors or angiotensin II receptor blockers, beta blockers, mineralocorticoid receptor antagonists, ivabradine or others were individualized according to the patient condition.
  (c) **Dapagliflozin**: It was given in a dose of 10 mg once daily.
  (d) **Insulin**: Insulin therapy was initiated if the blood glucose level was ≥180 mg/dL (10 mmol/L) after initiation of dapagliflozin treatment. Once insulin therapy was started, a target glucose range of 140–180 mg/dL (7.8–10 mmol/L) was recommended. Subcutaneous regular insulin every 6 h was used according to blood glucose level (22).
(B) *Group II (Control arm)*: The patients received:
  (a) **Furosemide**: as in study arm.
  (b) **Anti-failure treatment**: as in study arm.
  (c) **Insulin**: as in study arm.

All the patients underwent:

1. Continuous monitoring: Oxygen saturation and blood pressure monitoring.
2. Electrocardiogram (ECG) on admission and daily.
3. Complete echocardiographic assessment.
4. Laboratory assessment: including complete blood count, blood urea, serum creatinine, blood sugar, electrolytes, complete liver function tests on admission.
5. Follow up lab assessment: including blood sugar and urea, serum creatinine, sodium (Na^+^) and potassium (K^+^) were measured daily along the whole days of admission.

### Follow Up Parameters

#### Diuresis Parameters

- Total urine output: 24-h diuresis was quantified from admission till discharge and recorded in liters.
- Total fluid intake was calculated for patients and recorded in liters.
- Fluid balance was defined as the difference between total fluid intake and total urine output in liters.
- Fluid loss/diuretic: This relates the total urine output to the amount of administered furosemide in ml/mg.
- Fluid balance/diuretic: This relates the change in fluid balance to the amount of administered furosemide in ml/mg.
- Daily dose of furosemide and total dose of furosemide along whole hospital stay in mg were reported.
- Diuretic response: It was calculated according to Valente et al. (23). Forty milligrams Furosemide equal one diuretic unit. Diuretic response was calculated as change in body weight on the 4th day divided per diuretic units administered during days 1–3 (Δ in Kg/40 mg Furosemide) (23).

#### Changes in Body Weight Measurements

The body weight difference between admission and discharge was recorded in Kg. Also, the percentage of weight loss related to the initial body weight was reported. BMI (body mass index) and percentage of its change were recorded as well.

#### Dose of Insulin

The total dose of insulin used during the admission in order to control the blood sugar level was reported for both study arms in international units (IU).

#### Renal Function

It was determined every 24 h (during hospitalization) from admission till discharge. Renal function was assessed with the serum creatinine level. A WRF is defined as an increase ≥0.3 mg/dL in the serum creatinine level compared with the value on admission (24, 25).

#### Electrolyte Levels

Serum Na^+^ and K^+^ were assessed every 24 h (during hospitalization) from admission till discharge.

#### Patient-Reported Dyspnea

Patient-reported dyspnea was assessed every 24 h (during hospitalization) from admission till discharge.

Patient-reported dyspnea was assessed with the use of a five-point Likert scale (5PLS), a psychometric instrument for the measurement and grading of dyspnea (26–28). Many authors had validated this score and recommended its use to assess patients with ADHF (29, 30).

The scale includes the absence of dyspnea (a score of 1), mild shortness of breath (a score of 2), moderate shortness of breath (a score of 3), severe shortness of breath (a score of 4) and the worst possible shortness of breath (a score of 5). All patients filled out the 5PLS without any interference after a brief explanation provided by a nurse.

### Statistical Analysis

Data were verified, coded by the researcher, and analyzed using IBM-SPSS 21.0 (IBM-SPSS Inc., Chicago, IL, USA). Descriptive statistics: Means, standard deviations, and percentages were calculated. Test of significances: a Chi square test was used to compare the difference in distribution of frequencies among different groups, while for repeated measures (on admission vs. on discharge) the McNemar's test was used. A Student *t*-test analysis was carried out to compare the means of dichotomous data that follow the normal distribution. For repeated measures (on admission vs. on discharge) a paired sample *t*-test was used. It was considered as significant if the *p*-value was equal or <0.05.

## Results

This multi-center randomized clinical trial was conducted in the Cardiovascular Medicine Department, Assiut University Heart Hospital, Assiut University and Cardiology Department, Aswan University Hospital, Aswan University during the period from April 2020 to June 2020. This study involved 100 DM type 2 patients admitted with decompensated HF. The study cohort was randomly assigned to one of the two treatment modalities; 50 patients received dapagliflozin plus insulin (if needed) and furosemide plus conventional anti-HF measures (Study Group) and 50 patients received insulin plus furosemide and conventional anti-failure measures (Control group).

The two study groups were age and sex matched. There was no statistically significant difference between the two groups regarding history of hypertension, duration of diabetes mellitus, O_2_ saturation, blood pressure on admission as well as baseline anti-failure pharmacologic treatment. Table 1 showed the baseline characteristics of the study population.

**Table 1:** Baseline characteristics of the studied population.

**Parameter**	**Control Group (*n* = 50)**	**Study Group (*n* = 50)**	***P*-value**
Age in years (mean ± SD)	60.64 ± 9.9	62.02 ± 8.8	0.462^*^
Sex, male (%)	26 (52%)	28 (56%)	0.688^**^
Hypertensive (%)	31 (62%)	28 (56%)	0.542^**^
Duration of DM in years (mean ± SD)	13.04 ± 1.2	13.34 ± 1.1	0.875^*^
Weight in kg on admission (mean ± SD)	82.07 ± 9.1	80.56 ± 6.2	0.334^*^
BMI on admission (mean ± SD)	28.23 ± 3.3	27.78 ± 2.3	0.436^*^
Dyspnea on admission			
• Severe	11 (22%)	8 (16%)	0.444^**^
• Very Severe	39 (78%)	42 (84%)	
Serum creatinine on admission in μmol/L (mean ± SD)	1.40 ± 0.3	1.32 ± 0.2	0.126^*^
Serum Na^+^ on admission in mEq/L (mean ± SD)	137.64 ± 3.9	136.76 ± 3.6	0.241^*^
Serum K^+^ on admission in mEq/L (mean ± SD)	4.27 ± 0.6	4.18 ± 0.62	0.427^*^
RBS in mg/dL (mean ± SD)	272.16 ± 77.6	263.26 ± 83.1	0.583^*^
HbA1c in % (mean ± SD)	9.09 ± 2.1	8.61 ± 1.2	0.176^*^
O_2_ saturation in % (mean ± SD)	96.74 ± 2.05	97.02 ± 1.97	0.49^*^
Blood Pressure			
• Systolic blood pressure in mmHg (mean ± SD)	113.08 ± 14.97	110.74 ± 12.51	0.40^*^
• Diastolic blood pressure in mmHg (mean ± SD)	73.52 ± 9.29	72.88 ± 8.05	0.71^*^
Ejection Fraction in % (mean ± SD)	32.23 ± 2.49	32.54 ± 2.99	0.58^*^

* *Independent t-test test was used to compare the mean difference between groups*.

** *Chi-square test was used to compare proportions between groups*.

### Follow Up Parameters

#### Diuresis Parameters

Although the difference in fluid intake between the two groups was statistically insignificant, the amount of urine output was higher in the study vs. control groups (*p* < 0.001). Patients of the study group had higher fluid loss/diuretics (34.8 ± 2.21) compared to the controls (19.5 ± 1.23). Moreover, fluid balance/diuretics was significantly lower for the study (−21) compared with the control (−10) group (*p* < 0.01). The mean total dose of furosemide and furosemide dose/day were significantly lower for the study group compared with the control group (*p* < 0.01). The calculated diuretic response was more obvious among the study group (−0.089 ± 0.04) compared to the control group (−0.042 ± 0.03), *p*-value < 0.001, Table 2.

**Table 2:** Change associated with diuresis in the studied population.

**Parameter**	**Control Group (*n* = 50)**	**Study Group (*n* = 50)**	*P*-value^*^
Urine output in liters (mean ± SD)	14.43 ± 0.7	18.46 ± 0.5	<0.001^*^
Fluid intake in liters (mean ± SD)	7.01 ± 0.3	7.52 ± 0.2	0.139^*^
Total fluid balance in liters (mean ± SD)	−7.42 ± 0.7	−10.94 ± 0.4	<0.001^*^
Fluid loss/diuretics in ml/mg (mean ± SD)	19.49 ± 1.2	34.75 ± 2.2	<0.001^*^
Fluid balance/diuretics ml/mg (mean ± SD)	−9.87 ± 0.6	−20.86 ± 1.0	<0.001^*^
**Furosemide use**			
Total dose in mg (mean ± SD)	855.00 ± 74.8	597.60 ± 34.4	0.002^*^
Dose/day (mean ± SD)	170.78 ± 9.7	126.07 ± 4.3	<0.001^*^
Diuretic response in Kg/40mg furosemide (mean ± SD)	−0.042 ± 0.03	−0.089 ± 0.04	<0.001^*^

* *Independent t-test test was used to compare the mean difference between groups*.

#### Change in Body Weight Measurements

**Figure 2:**
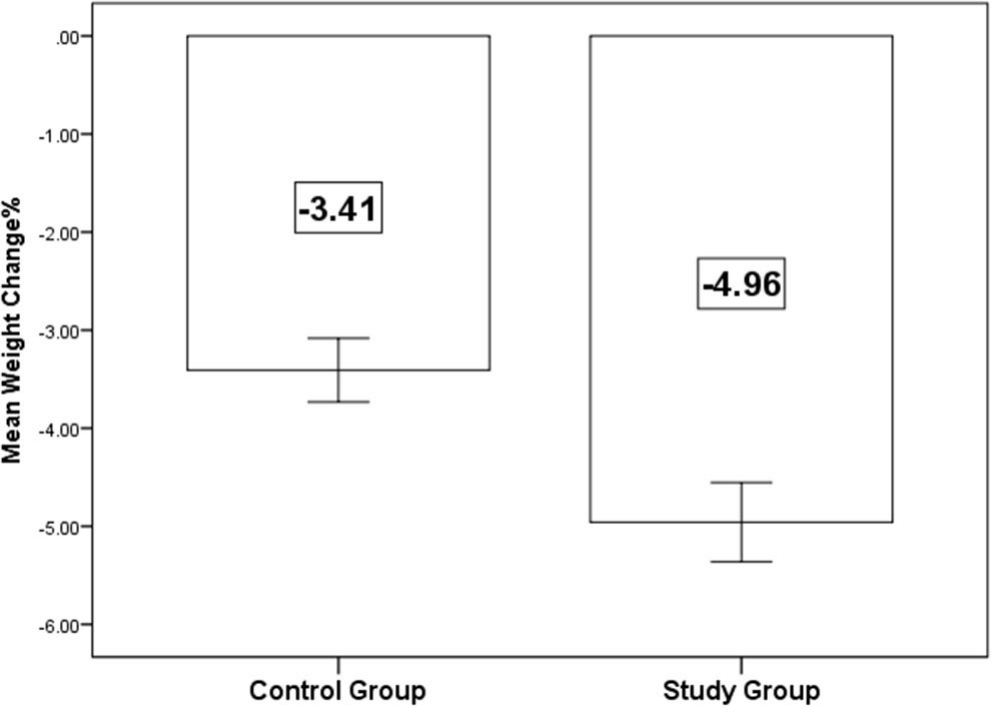
Mean percent change in the body weight on discharge.

Both groups showed no significant difference regarding mean values of weight and BMI on admission. On the other hand, on discharge, mean weight and BMI were lower in the study group (76.5 kg and 26.4) compared with the control (79.6 kg, 27.4) group (*p* = 0.004 and 0.074, respectively). The percent change for both measures was significantly higher (*p* < 0.001) for the study group (5%) compared with the controls (3.4%), Table 3 and Figure 2.

**Table 3:** Change in the BMI of the studied population.

**Parameter**	**Control Group (*n* = 50)**	**Study Group (*n* = 50)**	*P*-value^*^
Weight in Kg (mean ± SD)			
∂ On admission	82.07 ± 9.1	80.56 ± 6.2	0.334^*^
∂ On discharge	79.63 ± 8.9	76.51 ± 6.0	0.046^*^
*P*-value^**^	<0.001	<0.001	
Weight % change (mean ± SD)	−3.41 ± 0.2	−4.96 ± 0.2	<0.001^*^
BMI (mean ± SD)			
∂ On admission	28.23 ± 3.3	27.78 ± 2.3	0.436^*^
∂ On discharge	27.38 ± 3.1	26.39 ± 2.2	0.074^*^
*P*-value^**^	<0.001	<0.001	
BMI % change (mean ± SD)	−3.41 ± 0.2	−4.97 ± 0.2	<0.001^*^

* *Independent t-test test was used to compare the mean difference between groups*.

** *Paired t-test test was used to compare the mean difference between groups*.

#### Dose of Insulin

The total daily dose of insulin was significantly lower for the study group (29.6 ± 9.51 IU) compared with the control group (44.0 ± 13.33 IU) (*p* < 0.01).

#### Change in Renal Function and Serum Electrolytes

Both groups showed no significant difference regarding mean level of serum creatinine on admission. On the other hand, on discharge, mean level of serum creatinine was lower in the study group (1.39 ± 0.23 mg/dl) compared with the control group (1.53 ± 0.34 mg/dl) (*p* = 0.009). Significant increase in serum creatinine for both groups was observed on discharge (*p* < 0.01). There was a tendency of WRF to be more evident among the control group (28%) than the study group (16%), however, this difference did not reach a statistically significant level, *p*-value 0.148, Table 4.

**Table 4:** Change in renal function and serum electrolytes of the studied population.

**Parameter**	**Control Group (*n* = 50)**	**Study Group (*n* = 50)**	*P*-value^*^
**Serum creatinine level in μmol/L (mean ± SD)**			
∂ On admission	1.40 ± 0.3	1.32 ± 0.2	0.126^*^
∂ On discharge	1.53 ± 0.3	1.39 ± 0.2	0.009^*^
*P*-value^**^	<0.001	0.003	
Serum creatinine level % change (mean ± SD)	12.34 ± 2.9	8.76 ± 2.5	0.349^*^
WRF (%)	14 (28)	8 (16)	0.148^**^
**Serum Na**^**+**^ **level in mEq/L (mean ± SD)**			
∂ On admission	137.64 ± 3.9	136.76 ± 3.6	0.24^*^
∂ On discharge	131.52 ± 3.2	131.96 ± 2.7	0.46^*^
*P*-value^**^	<0.001	<0.001	
S. Na^+^ Level % change (mean ± SD)	4.42 ± 2.0	3.48 ± 1.70	0.01^*^
**Serum K**^**+**^ **level in mEq/L (mean ± SD)**			
∂ On admission	4.27 ± 0.6	4.18 ± 0.6	0.427^*^
∂ On discharge	3.83 ± 0.5	4.11 ± 0.4	0.003^*^
*P*-value^**^	<0.001	0.005	
S. K^+^ Level % change (mean ± SD)	9.82 ± 0.9	1.37 ± 0.7	<0.001^*^

* *Independent t-test test was used to compare the mean difference between groups*.

** *Paired t-test test was used to compare the mean difference between groups*.

Likewise, both groups showed no significant difference regarding mean K^+^ level on admission. Contrarily, on discharge, mean level of serum K^+^ was higher in the study group (4.11 ± 0.42 mEq/L) compared with the control group (3.83 ± 0.50 mEq/L) (*p* = 0.003).

On the other hand, serum sodium level was comparable between the two study arms on admission and on discharge levels. However, overall, there was statistically significant reduction in serum sodium for both groups on discharge (*p* < 0.001), Table 4.

#### Patient-Reported Dyspnea

**Figure 3:**
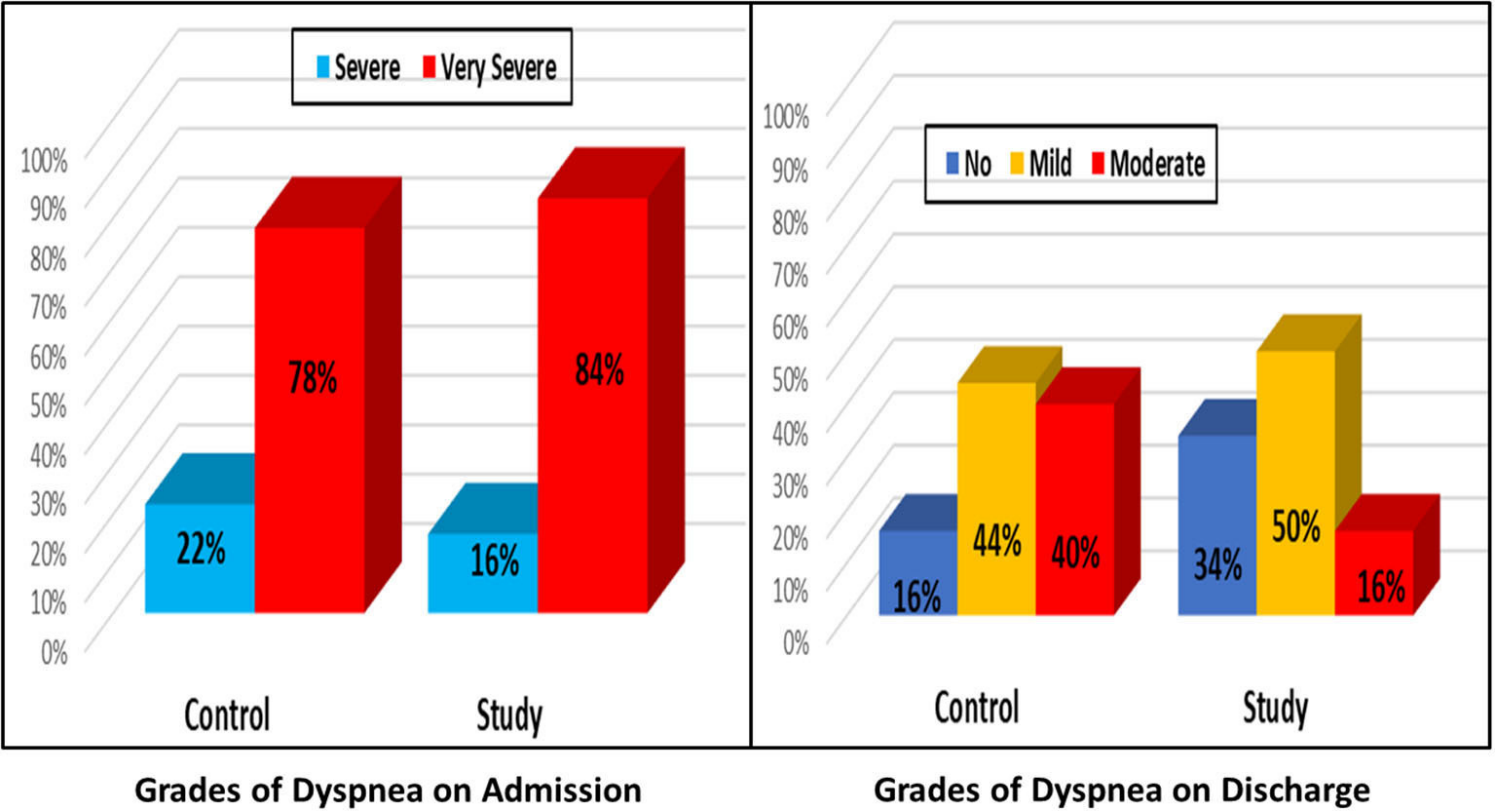
Change in the dyspnea grades on admission vs on discharge.

At baseline, in both groups about one-fifth (22 vs. 16%) of patients had severe dyspnea and the other four-fifths had very severe grades (78 vs. 84%) (*p* = 0.444). On discharge, the study group had better improvement [about one third had no dyspnea (34%), about one half had mild grade and only 16% had moderate grade] compared to the control group (about 16% had no dyspnea, 44% had mild and 40% had moderate grade of dyspnea) (*p* < 0.002). Overall, significant improvement was observed on discharge for both groups (*p* < 0.001), Figure 3.

#### Other Outcomes

There was no statistically significant difference between the two study arms regarding mortality during hospitalization, one case in each arm. There was also no statistically significant difference between the two arms regarding the duration of hospital stay, 4.92 ± 1.52 days for the control group vs. 4.64 ± 1.01 days for the study group, *p*-value 0.27.

## Discussion

Type 2 diabetes mellitus (T2DM) was associated with increased incidence of congestive heart failure (CHF) (31, 32). Congestion in heart failure is defined as manifestations of extracellular fluid expansion that results in increased cardiac filling pressures (33). HF with increased neurohumoral activation leads to increased renal avidity to sodium and water, hence an increased plasma volume (34, 35). Increased sympathetic output also leads to splanchnic arterial and venous constriction and blood redistribution from the splanchnic capacitance vasculature to the circulatory volume. This increases the effective circulating volume by redistribution, in a state where volume expansion is already present (36).

Loop diuretics are the corner stone of treatment for patients with ADHF and fluid overload (37). However, many patients show a poor response, with up to 50% considered to be diuretic resistant (23). Prolonged administration of loop diuretics increases Na^+^ reabsorption at the distal nephron segments, thereby limiting Na^+^ loss (38, 39). This “diuretic braking phenomenon” (40) definitely leaves many patients with CHF with an expanded blood volume that predicts adverse outcomes (41).

In high doses, diuretics activate the RAAS and may promote HF progression (42, 43). Furthermore, excess diuretics causes plasma volume contraction, WRF and leads to various electrolyte disturbances including hypokalemia, hypomagnesemia, hypocalcemia, hyponatremia, and hyperuricemia (44–46). While mineralocorticoid receptor antagonists (MRAs) have mild diuretic effects and improve prognosis in HF with reduced EF (47); hyperkalemia and WRF are common side effects of these drugs (48).

Although originally developed as glucose-lowering medications for patients with T2DM, SGLT-2 inhibitors have improved event-free survival in patients with chronic HF, regardless of the degree of hyperglycemia or diabetic status (16, 49). SGLT-2 inhibitors increase urinary excretion of glucose and sodium and appear to produce a durable reduction in blood volume (50, 51). SGLT-2 accounts for a portion of proximal Na^+^ reabsorption (52, 53). Its inhibition causes an osmotic diuresis that can enhance Na^+^ excretion (54). However, unlike traditional diuretics, their action involves limited activation of the neurohormonal system and insignificant changes in the electrolyte profile of the patient (55).

Reports from the EMPA-REG OUTCOME and the CANVAS showed that SGLT-2 inhibitors were effective for medium- and long-term inhibition of major adverse cardiovascular events and the progression of renal dysfunction (16, 17). In the placebo-controlled Dapagliflozin And Prevention of Adverse-outcomes in Heart Failure (DAPA-HF) trial, dapagliflozin reduced the risk of HF hospitalization and mortality, and improved symptoms, in more than 4,500 patients with heart failure and reduced ejection fraction (HFrEF) (19, 56).

Therefore, SGLT-2 inhibitors may be a good option in patients with T2DM and CHF, the interaction between SGLT-2 inhibitors and furosemide needs a well randomized prospective study. An augmented natriuresis with one diuretic when added to the other would indicate a synergetic effect, such as that shown with loop diuretics and thiazides (39). This study tested the hypothesis that there would be favored interactions between these two classes of drugs (dapagliflozin and furosemide) in patients with T2DM and ADHF and, to our knowledge, this is the first prospective randomized controlled trial to test the effect of both agents when given together in patients with ADHF.

In 2020 Petrie et al. evaluated the effects of dapagliflozin in patients with HFrEF with and without diabetes, where 10 mg of dapagliflozin or a placebo were added to the recommended therapy once-daily. They concluded that dapagliflozin significantly reduced the risk of worsening HF or CV death independently of diabetes status (57).

The diuretic actions of SGLT-2 inhibitors presumably play an important role in cardioprotection, as shown in the EMPA-REG OUTCOME study and the CANVAS program. SGLT-2 inhibitors have acutely caused an increase in urinary sodium excretion in non-diabetic (58) and diabetic rats (59, 60). Our study showed that addition of dapagliflozin to furosemide actually improved all studied diuresis parameters including urine output, total fluid balance as well as fluid balance/diuretic dose. In a small randomized, placebo-controlled, double-blind trial, involving 75 subjects with T2DM, dapagliflozin has been shown to reduce plasma volume in a similar way to thiazide diuretics, but dapagliflozin has a more enduring diuretic effect than other diuretics (50).

In 2018, Wilcox et al. (61) concluded that first-dose Na^+^ excretion with bumetanide and dapagliflozin is not additive, but the weekly administration of one diuretic enhances the initial Na^+^ excretion with the other. Thus, there was a significant two-way adaptive natriuretic synergy. This resulted in a greater Na^+^ excretion during the second week when both diuretics were given together. Prior diuretic administration was required to evoke this synergistic natriuretic interaction (61). If we assume that this postulation was correct, this would explain the rapid and good response for combined therapy with both dapagliflozin and furosemide in our enrolled patients as one of our prerequisites to include patients was that the patient should already be on furosemide for at least 1 month before admission.

Our results reported a statistically significant reduction in serum sodium for both study arms. However, the percentage reduction in serum Na^+^ was significant for the control arm (4.4% for control group vs. 3.5% for the study group, *p*-value 0.01). The control group received relatively large doses of furosemide (mean total furosemide dose was 855 mg in control group vs. 597 mg in study group). Despite the fact that the study reported an obvious improvement in all studied diuresis parameters, we did not notice any deleterious effects of dapagliflozin on serum K^+^. The use of dapagliflozin was not associated with hypokalemia or WRF as observed with diuretics alone. The hypothesis that the use of dapagliflozin acutely reduced the dose of needed furosemide hence limiting its associated side effects including hypokalemia and renal troubles. In agreement with our results, the retrospective analysis done by Griffin et al. (62) showed that therapy with an SGLT-2 inhibitor was associated with improved urine output and weight loss after therapy. These effects were observed without increase of loop diuretic or thiazide therapy, and the resultant diuretic efficiency was markedly improved as daily urine output improved during Day 1 (*P* = 0.002), Day 2 (*P* = 0.02), and Day 3 (*P* = 0.02) compared with the 24 h prior to treatment. They also detected no adverse outcomes, including deterioration of renal function, change in blood pressure or electrolytes, or genitourinary infections while on therapy (62). Regarding safety of using dapagliflozin in patients with HF, our results go hand in hand with DAPA-HF findings which revealed that the beneficial effects of dapagliflozin was not associated with any adverse events on renal function. (19). Cahn et al. also confirmed that SGLT-2 inhibitors do not increase risk for acute kidney injury compared with Dipeptidyl peptidase-4 (DPP-4) inhibitors among patients with T2DM (63).

In concordance with our results concerning change in potassium level, Yavin et al. found that dapagliflozin did not appear to increase serum K^+^ levels in patients with T2DM, including patients at a higher risk of hyperkalemia, such as those with moderate renal impairment or treated with angiotensin converting enzymes (ACE) inhibitors, angiotensin II receptor blockers (ARBs), or potassium-sparing diuretics (64). Although, Wilcox et al. agreed with our results as they showed that there were no clinically significant changes in serum sodium, or creatinine concentrations. They found that dapagliflozin induced hypokalemia with bumetanide. Serum K^+^ was unchanged by dapagliflozin alone but was reduced 7% by bumetanide alone and 12% by the combination, reflecting increases in renal K^+^ excretion. They explained the greater K^+^ excretion and hypokalemia with combined therapy as a consequence of hyperaldosteronism because there were high levels of plasma renin activity (61).

In our study, the use of dapagliflozin has reduced the mean total dose of required furosemide by approximately one third (mean total furosemide dose was 855 mg in control group vs. 597 mg in study group). A similar pattern of observations was obtained by Kambara et al. who concluded that the use of SGLT-2 inhibitors (empagliflozin and canagliflozin) was safe and effective in DM patients who required inpatient treatment for acute HF. Early initiation of SGLT-2 inhibitor therapy after the onset of acute HF reduced the doses of loop diuretics (to approximately one third), leading to greater prevention of acute kidney injury (65). It is worth noting that his study was a retrospective, and was not randomized and the sample size was relatively small, including only 31 patients (12 patients in SGLT-2 inhibitor group and 19 patients in the conventional treatment group). None of the patients received dapagliflozin and nine patients (75%) received empagliflozin and three patients (25%) received canagliflozin (65).

Subgroup analysis from the DAPA-HF trial was carried out by Jackson et al. (66). They examined a dapagliflozin effect in the following subgroups: no diuretic and diuretic dose equivalent to furosemide <40, 40, and >40 mg daily at baseline. The benefit of dapagliflozin was clear regardless of background diuretic therapy and across the range of background doses of diuretic used in DAPA-HF. The analysis also proved the tolerability and safety of dapagliflozin in patients who were treated with a standard diuretic or not. The mean dose of furosemide did not differ between the dapagliflozin and placebo group during follow-up. Most patients did not change their diuretic dose. A small proportion had changed the diuretic dose—an increase was less likely while a decrease was more likely in the dapagliflozin arm compared with the placebo arm (66).

As the addition of dapagliflozin ensured more diuresis, our study detected a statistically significant difference regarding the percentage of change in the body weight (3.4 kg for control arm vs. 5 kg for the study arm; *p*-value 0.001). The effects of empagliflozin on cardiorespiratory fitness in patients with T2DM and HFrEF were studied by Carbone et al. Empagliflozin reduced body weight (−1.7 kg; *P* = 0.031) but did not change peak oxygen consumption. However, patients using loop diuretics (*n* = 9) demonstrated an improvement, whereas those without loop diuretics (*n* = 6) experienced a decrease in peak oxygen consumption and peak oxygen consumption changes correlated with the baseline daily dose of diuretics (*R* = +0.83; *P* < 0.001) (21). The most important finding would be that the use of empagliflozin in HFrEF patients not treated with loop diuretics may be less beneficial and this could greatly influence the final therapeutic outcome (21).

In our study, the use of dapagliflozin was associated with dyspnea improvement, which was more pronounced than that associated with the diuretic alone. Dyspnea improvement in HF patients is mostly attributed to reduction in plasma volume that can be carried out effectively by diuretics, especially loop diuretics. However, to achieve a good reduction of plasma volume, we may be forced to use high doses of diuretics and this is mostly associated with side effects such electrolyte imbalance. This electrolyte imbalance can cause muscle fatigue especially the respiratory muscles, hence the continued sense of dyspnea. This could be the case in the control arm of our study where we used large doses of furosemide. On the other hand, in the study arm, the reduction of plasma volume was achieved by the synergistic effect of using dapagliflozin and furosemide in relatively lower doses than the control arm, so less side effects, less muscle fatigue and less dyspnea. Incongruency with our findings, in 2020, Damman et al. (67) found that in patients with acute HF, treatment with empagliflozin had no effect on change in visual analog scale, dyspnea score, diuretic response, N-terminal pro-natriuretic peptide (NT-pro BNP), and length of hospital stay, but was safe, increased urinary output, and reduced endpoint of worsening HF, re-hospitalization for HF, or death at 60 days (67).

Of course, our study had some limitations. Despite meaningful effects which were extrapolated from our study with respect to the synergetic effect of adding dapagliflozin to furosemide in patients with ADHF. It was difficult to clarify whether there was a remarkable interaction with other anti-failure drugs or not. A second limitation was that the only loop diuretic which was used in our study is furosemide so further research is clearly required to ascertain such synergetic effects with other loop diuretics. Further studies that design dapagliflozin and furosemide as a long-term treatment for HF patients are needed for a better assessment of this combination therapy for such patients. Lastly, serial assessment of heart failure biomarkers such as BNP would be of great value to this work, as it would help to better assess dyspnea improvement in both study groups. However, unfortunately, biomarkers like BNP have not been routinely assessed.

## Conclusions

Dapagliflozin is a relatively newly introduced anti-diabetic drug, however, it demonstrates outstanding diuretic effects that put it among the lines of treatment used for HF in DM patients. Its use potentiates the action of loop diuretics and lowers their dose. It has a non-remarkable effect on serum potassium and renal function.

## Data Availability Statement

The original contributions presented in the study are included in the article/Supplementary Materials, further inquiries can be directed to the corresponding author/s.

## Ethics Statement

The studies involving human participants were reviewed and approved by Ethical Committee of the Faculty of Medicine, Assiut University. The patients/participants provided their written informed consent to participate in this study.

## Author Contributions

AI and AA-G: concept, design, literature search, clinical studies, experimental studies manuscript preparation, editing, and review. RG, HM, AH, NM, MA, SE, and ME: definition of intellectual content, literature search and manuscript review, clinical studies, experimental studies, and data acquisition. LA-W: clinical studies, experimental studies, and data acquisition. AKI: data analysis, statistical analysis, manuscript preparation, editing and review. All authors contributed to the article and approved the submitted version.

## Conflict of Interest

The authors declare that the research was conducted in the absence of any commercial or financial relationships that could be construed as a potential conflict of interest.

## Abbreviations

ADHF: Acute Decompensated Heart Failure
HF: Heart Failure
WRF: Worsening Renal Function
DM: Diabetes Mellitus
ASCVD: Atherosclerotic cardiovascular disease
SAVOR-TIMI 53: Saxagliptin Assessment of Vascular Outcomes Recorded in Patients with DM-TIMI-53
EMPA-REG OUTCOME: The Empagliflozin Cardiovascular Outcome Event Trial in Type 2 Diabetes Mellitus Patients–Removing Excess Glucose (EMPA-REG) OUTCOME trial
CV: Cardiovascular
CANVAS: Canagliflozin Cardiovascular Assessment Study
CANVAS-R: Canagliflozin Cardiovascular Assessment Study-Renal
DECLARE-TIMI 58: Dapagliflozin Effect on Cardiovascular Events-Thrombolysis in Myocardial Infarction 58 trial
SGLT2: Sodium-glucose co-transporter-2
EF: Ejection fraction
RAAS: Renin-angiotensin-aldosterone system
LVEF: Left ventricular ejection fraction
GFR: Glomerular filtration rate
ECG: Electrocardiogram
Na^+^: Sodium
K^+^: Potassium
BMI: Body mass index
IU: International unit
5PLS: Five-point Likert scale
T2DM: Type 2 diabetes mellitus
CHF: Congestive heart failure
MRAs: Mineralocorticoid receptor antagonists
DAPA-HF: Dapagliflozin And Prevention of Adverse-outcomes in Heart Failure
HFrEF: Heart failure with reduced ejection fraction
DPP-4: Dipeptidyl peptidase-4
ACE: Angiotensin converting enzyme
ARBs: Angiotensin II receptor blockers
NT-pro BNP: N-terminal pro-natriuretic peptide.
