# Supplementary material for: Safety and Efficacy of Adding Dapagliflozin to Furosemide in Type 2 Diabetic Patients With Decompensated Heart Failure and Reduced Ejection Fraction
Source: Front Cardiovasc Med. 2020 Dec 7;7:602251. doi: 10.3389/fcvm.2020.602251 (PMC7793915; doi:10.3389/fcvm.2020.602251)
Supplement: Supplementary file 1 [file Data_Sheet_1.PDF]

**Supplementary Table 1: Baseline characteristics of the studied population**

| Parameter | Control Group (n=50) | Study Group (n=50) | P-value |
|---|---|---|---|
| Age in years (mean ± SD) | 60.64 ± 9.90 | 62.02 ± 8.81 | 0.462* |
| Sex, male (%) | 26 (52%) | 28 (56%) | 0.688** |
| Hypertensive (%) | 31 (62%) | 28 (56%) | 0.542** |
| Duration of DM in years (mean ± SD) | 13.04 ± 1.22 | 13.34 ± 1.13 | 0.875* |
| Weight/kg on admission (mean ± SD) | 82.07 ± 9.10 | 80.56 ± 6.21 | 0.334* |
| BMI on admission (mean ± SD) | 28.23 ± 3.32 | 27.78 ± 2.33 | 0.436* |
| Dyspnea on admission | | | |
| • Severe | 11 (22%) | 8 (16%) | 0.444** |
| • Very Severe | 39 (78%) | 42 (84%) | |
| Serum creatinine on admission (mean ± SD) | 1.40 ± 0.32 | 1.32 ± 0.24 | 0.126* |
| Serum Na on admission in mEq/L (mean ± SD) | 137.64 ± 3.93 | 136.76 ± 3.64 | 0.241* |
| Serum K on admission in mEq/L (mean ± SD) | 4.27 ± 0.63 | 4.18 ± 0.62 | 0.427* |
| RBS (mean ± SD) | 272.16 ± 77.60 | 263.26 ± 83.11 | 0.583* |
| HbA1c (mean ± SD) | 9.09 ± 2.12 | 8.61 ± 1.24 | 0.176* |
| $O_2$ saturation | 96.74 ± 2.05 | 97.02 ± 1.97 | 0.49* |
| SBP | 113.08 ± 14.97 | 110.74 ± 12.51 | 0.40* |
| DBP | 73.52 ± 9.29 | 72.88 ± 8.05 | 0.71* |
| Echocardiographic data: | | | |
| • LVEDD | 6.31 ± 0.57 | 6.23 ± 0.51 | 0.47* |
| • LVESD | 5.24 ± 0.46 | 5.17 ± 0.45 | 0.43* |
| • FS | 16.97 ± 1.31 | 16.88 ± 2.41 | 0.83* |
| • EF | 32.23 ± 2.49 | 32.54 ± 2.99 | 0.58* |

| | | | |
|---|---|---|---|
| • Mitral regurge | | | 0.53** |
| - Grade I | 8 (16) | 4 (8) | |
| - Grade II | 19 (38) | 17 (34) | |
| - Grade III | 15 (30) | 18 (36) | |
| - Grade IV | 8 (16) | 11 (22) | |
| Pharmacologic treatment: | | | |
| • ACE-I | 33 (66) | 37 (74) | 0.38** |
| • ARBs | 12 (24) | 9 (18) | 0.46** |
| • ARNI | 2 (4) | 4 (8) | 0.40** |
| • Beta-Blockers | 2 (4) | 3 (6) | 0.65** |
| • Digoxine | 20 (40) | 18 (36) | 0.68** |
| • MRAs | 43 (86) | 41 (82) | 0.59** |
| • Thiazide | 4 (8) | 5 (10) | 0.73** |

* Independent t-test test was used to compare the mean difference between groups
** Chi-square test was used to compare proportions between groups
